# Kinetics of drug action in disease states: towards physiology-based pharmacodynamic (PBPD) models

**DOI:** 10.1007/s10928-015-9437-x

**Published:** 2015-08-30

**Authors:** Meindert Danhof

**Affiliations:** Leiden Academic Centre for Drug Research, Leiden University, P.O. Box 9502, 2300 RA Leiden, The Netherlands

**Keywords:** Biophase distribution, Receptor theory, Dynamical systems analysis, Disease systems analysis

## Abstract

Gerhard Levy started his investigations on the “Kinetics of Drug Action in Disease States” in the fall of 1980. The objective of his research was to study inter-individual variation in pharmacodynamics. To this end, theoretical concepts and experimental approaches were introduced, which enabled assessment of the changes in pharmacodynamics per se, while excluding or accounting for the cofounding effects of concomitant changes in pharmacokinetics. These concepts were applied in several studies. The results, which were published in 45 papers in the years 1984–1994, showed considerable variation in pharmacodynamics. These initial studies on kinetics of drug action in disease states triggered further experimental research on the relations between pharmacokinetics and pharmacodynamics. Together with the concepts in Levy’s earlier publications “Kinetics of Pharmacologic Effects” (Clin Pharmacol Ther 7(3): 362–372, 1966) and “Kinetics of pharmacologic effects in man: the anticoagulant action of warfarin” (Clin Pharmacol Ther 10(1): 22–35, 1969), they form a significant impulse to the development of physiology-based pharmacodynamic (PBPD) modeling as novel discipline in the pharmaceutical sciences. This paper reviews Levy’s research on the “Kinetics of Drug Action in Disease States”. Next it addresses the significance of his research for the evolution of PBPD modeling as a scientific discipline. PBPD models contain specific expressions to characterize in a strictly quantitative manner processes on the causal path between exposure (in terms of concentration at the target site) and the drug effect (in terms of the change in biological function). Pertinent processes on the causal path are: (1) target site distribution, (2) target binding and activation and (3) transduction and homeostatic feedback.

## Introduction

Gerhard Levy started his investigations in the series “Kinetics of Drug Action in Disease States”, in the fall of 1980. At that time it was well-established that multiple factors including certain diseases, changes in physiology, concomitant use of other drugs and environmental factors can have profound effects on the pharmacokinetics of drugs [1]. Also, the awareness of large inter-individual variation in pharmacokinetics had led to the introduction of therapeutic drug concentration monitoring in clinical practice as the basis for individualized optimization of drug dosage, yielding plasma concentrations in a pre-defined therapeutic range [2–4]. In this practice it was implicitly assumed that inter-individual variation in pharmacodynamics is small. However, limited information was available on variation in pharmacodynamics (i.e. drug concentration–effect relations). A review of the literature revealed that there are many examples of altered drug response as result of disease, but that in general it was not possible to determine the mechanism of this altered response in terms of changes in pharmacokinetics, pharmacodynamics or a combination of both. In other words, the magnitude and the underlying mechanisms of inter-individual variation in pharmacodynamics were largely unknown. It were these observations that that made Levy decide to start systematic investigations on the variation in pharmacodynamics in animal models of disease [5]. It was the starting point of experimental research in pharmacodynamics. Initially, in this research the emphasis was on designing novel concepts and approaches by which, in pharmacodynamic studies, the effects of potentially confounding changes in pharmacokinetics (e.g. changes in distribution, accumulation of metabolites) can be either excluded or accounted for. Application of these approaches in conjunction with the concepts from Levy’s earlier papers on the kinetics of pharmacological effects [6] and the modeling of the anticoagulant actions of warfarin [7] led to fundamental insights in the relations between pharmacokinetics and pharmacodynamics. For example the concepts from Levy’s key publication “Kinetics of pharmacologic effects” [6] constituted the basis for the modeling of target site distribution as a determinant of the time course of drug effect. And the publication “Kinetics of pharmacologic effects in man: the anticoagulant action of warfarin” [7] constitutes the scientific basis for not only the modeling of time dependencies in drug action resulting from an indirect mechanism of action, but also the modeling of transduction mechanisms, of homeostatic feedback and even the modeling of drug effects on disease progression. Rigorous animal experiments, in which drugs were administered at widely different rates and routes of administration, in which drug and metabolite concentrations were measured in different tissues, and where quantitative information on relevant system properties such as the receptor density was obtained, constituted the basis for development of mechanism-based PKPD models with improved properties for extrapolation and prediction. These contributions represent a significant impulse to the development of physiology-based pharmacodynamic (PBPD) modeling as a novel discipline in the pharmaceutical sciences.

This paper reviews Levy’s research on the “Kinetics of Drug Action in Disease States”. Next it addresses the significance of his research for the evolution of PBPD modeling as a scientific discipline, focusing on models to characterize: (1) the kinetics of target site distribution, (2) the molecular mechanisms of variation in concentration–effect relations, (3) the kinetics of transduction and homeostatic feedback, and (4) the mechanism-based analysis of drug effects on disease progression.

## Separating pharmacokinetic and pharmacodynamic variability

Apparent changes in drug concentration–effect relationships in disease states can be the result of changes in pharmacokinetics, changes in pharmacodynamics, or both. Therefore, to investigate the pharmacodynamic variability in vivo, and to reveal the true magnitude of this variability, it is important to apply experimental approaches by which the effects of potentially confounding changes in pharmacokinetics are either excluded or accounted for. Potentially confounding pharmacokinetic factors include: (1) changes in distribution between the site where the drug concentrations are measured and the site of action (the “biophase”), (2) changes in the disposition of enantiomers, (3) the formation of active and/or interactive metabolites, and (4) functional adaptation or tolerance development resulting from variation in the duration of drug action. In this section these approaches and their applications are discussed and illustrated based on examples from the series on “Kinetics of Drug Action in Disease States”.

### Target site distribution kinetics

Understanding variation in pharmacodynamics requires information on the (free) drug concentration at the target site. For drugs acting at extracellular targets this information might be derived from the free drug concentrations in plasma by postulating a “effect compartment” to account for hysteresis as was elegantly demonstrated for d-tubocurarine by Sheiner et al. [8]. To what extent this also applied to other drugs which differ in target binding kinetics or for drugs acting in different tissues, was largely unknown. Moreover, for drugs acting in tissues that are protected by specific barriers (i.e. the central nervous system) or for drugs acting at intracellular targets (i.e. anti-cancer drugs), the biophase distribution kinetics are likely to be much more complex. Here changes in the permeability of the barriers and/or the expression and function of transporters may lead to apparently different plasma drug concentration–effect relationships, while the underlying target concentration–effect relation is unaltered. Or conversely, the free steady-state plasma concentration–effect relationship may seem unaffected despite a change in the underlying pharmacology. It was therefore deemed essential to develop approaches to approximate free drug concentration–effect relations to account for eventual disease related variation in target distribution kinetics. This started the research on identifying a compartment where drug and metabolite concentrations could be measured and which was, as we called it, “pharmacokinetically indistinguishable from the site of action” [5]. Here is a direct connection to Levy’s 1966 paper on the kinetics of pharmacological effects [6], where it is described how he had applied this principle to demonstrate that the effect of d-tubocurarine was related to concentrations in a peripheral compartment of a multi-compartment pharmacokinetic model rather than the concentrations in blood [9].

**Fig. 1:**
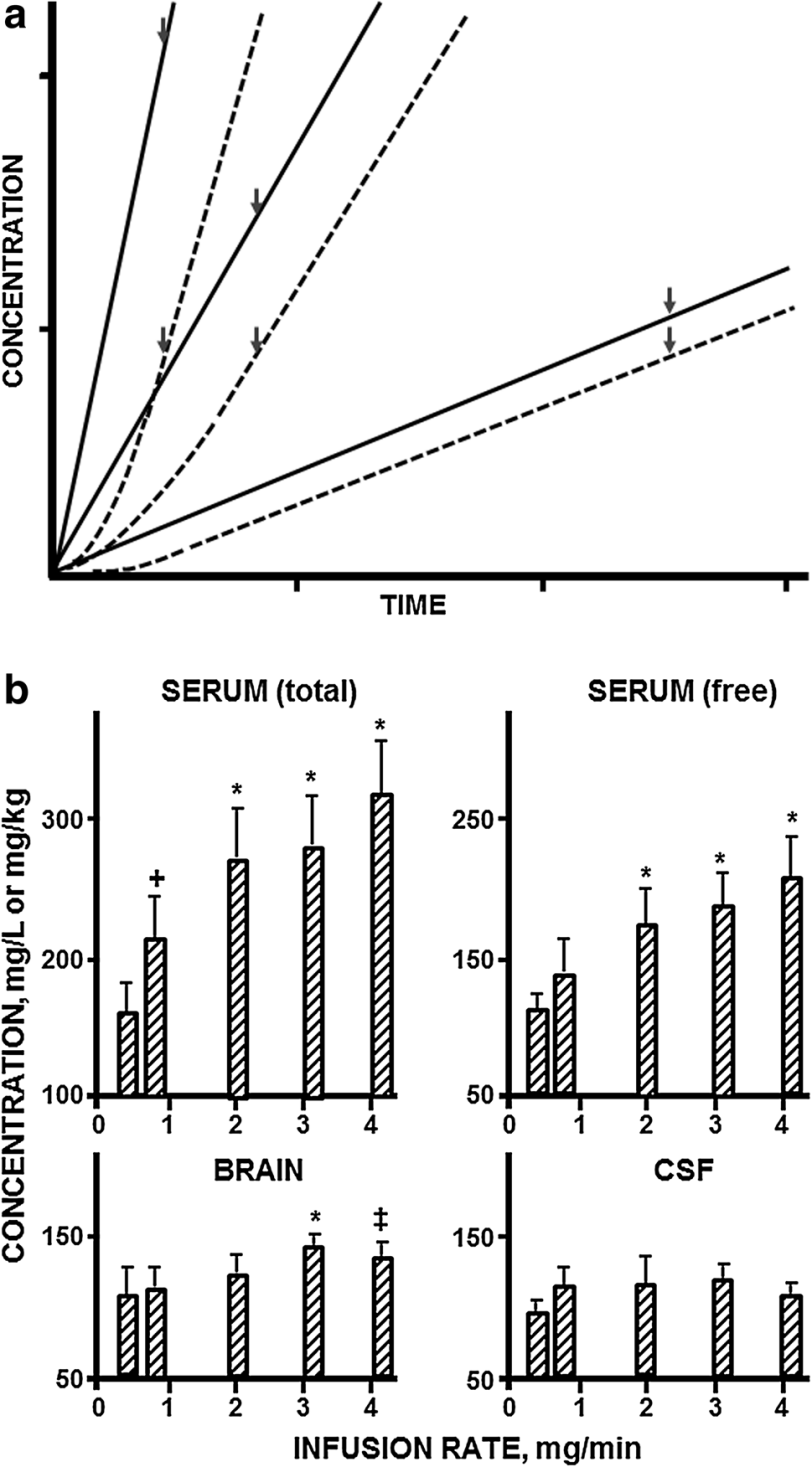
**a** Schematic representation of drug concentration versus time profiles at three different infusion rates in the plasma (*continuous lines*) and at the site of action (*dashed lines*). The time of onset of a pharmacologic effect is indicated by *arrows*. The representation is a simulation of a two-compartment system with a drug clearance of 0.029 l/h, a terminal drug half-life of 24 h and infusion rates of 0.42, 2.5 and 4.2 mg/min. It should be noted that the drug concentration in plasma at onset of effect decreases with decreasing infusion rate. **b** Effect of infusion rate on the concentration of phenobarbital in serum (total and unbound drug, respectively), brain and CSF of female rats at the onset of loss of righting reflex. Results are the mean of five to nine animals per group, with the *vertical line* indicating 1 SD. Infusion rate had a significant effect (p < 0.001 by one-way analysis of variance) on drug concentrations in serum and brain but not on concentrations in CSF. The *symbols* above the *vertical bars* indicate significant differences from the results produced by the lowest infusion rate (*p < 0.002; ^‡^p < 0.01; ^+^p < 0.05; Newman-Keuls test). Reproduced from Danhof and Levy 1984 [5]

In the first paper in the series on the kinetics of drug action in disease states, we introduced the concept of infusion of sedative drugs to a predefined degree of sedation (loss of righting reflex, LRR) to identify a site where drug concentrations were in direct equilibrium with the target site, to ultimately be able to study changes in the brain sensitivity to sedative and anesthetic drugs [5]. For this first study, phenobarbital was chosen as a model drug, because of its favorable pharmacokinetic properties, in that unlike many other barbiturates, phenobarbital is not an enantiomeric drug. Moreover, the drug is slowly metabolized and *p*-hydroxy-phenobarbital had been identified as its major metabolite, which was commercially available, and of which the effects could be studied. A specific feature of using drug concentrations at the onset of LRR as pharmacodynamic endpoint (rather than the more traditional approach of measuring the concentration at offset in a sleeping time experiment) is that the concentrations are obtained under disequilibrium conditions. This enables identification of the compartment which is indistinguishable from the site of action, which is the compartment where the free drug concentration at onset of LRR is independent of the rate of infusion (Fig. 1a). These studies showed that only in cerebrospinal fluid (CSF), but not in plasma or brain tissue, the phenobarbital concentration at onset of LRR is independent of the infusion rate. Thus, in this manner the CSF was identified as a compartment that, unlike blood plasma or whole brain tissue, is pharmacokinetically indistinguishable from the site of action (Fig. 1b) [5]. A further advantage of the use of CSF concentrations is that, due to the absence of significant protein concentrations, they reflect free drug concentrations in the brain which presumably are pharmacologically more relevant compared to total brain concentrations. In subsequent investigations from Levy’s laboratory and others these investigations were extended to different drugs and different pharmacodynamic endpoints (Table 1). For example studies on the sedative effects were extended to other barbiturates with different physicochemical properties(e.g. heptabarbital) and drugs with different molecular targets such as, ethanol [10] benzodiazepines (e.g. diazepam, oxazepam [11, 12]), zoxazolamine (and its metabolite chlorzoxazone, [13]), and salicylamide [14]. Next the studies were extended to other pharmacodynamic endpoints (e.g. convulsant effects of pentylenetetrazole, PTZ) [15, 16]. These studies showed that for most of the drugs studied, the CSF concentrations are uniquely representative for the target site concentrations, while for other drugs (salicylamide and PTZ) there is rapid equilibrium between the concentrations in plasma, brain tissue, and CSF making them equally useful for use in pharmacodynamic investigations (Table 1). The principles of identifying a compartment in which drug concentrations are in direct equilibrium with effect site concentrations, and the use of CSF in pharmacodynamic studies on CNS active drugs were adopted by other research groups [17–21]. This generated an interest in the use of CSF drug concentrations in investigations on the pharmacodynamics of CNS active drugs (for review see: [22]).

**Table 1:** Studies on the identification of a site where drug concentrations were in direct equilibrium with the target site

	Studies in which CSF concentrations were identified as the compartment ‘pharmacokinetically indistinguishable from the site of action’	Studies in which concentrations in serum, brain and CSF at onset of a defined pharmacologic effect were independent of infusion rate
CNS depressants Pharmacodynamic endpoints Onset of loss of righting reflex Offset of loss of righting reflex	Effect of infusion rate on *phenobarbital* concentrations in serum, brain and cerebrospinal fluid of normal rats at onset of loss of righting reflex [5] Pharmacodynamics of *diazepam* and its active metabolites in rats [11] Effect of repeated blood sampling on the pharmacodynamics of *phenobarbital* in rats [91]	Pharmacodynamics of the hypnotic effect of *salicylamide* in rats [14]^a^
CNS stimulants Pharmacodynamic endpoints Onset of seizures (first myoclonic jerk, twitch) Onset of maximal seizures (tonic flexion of the forelimbs and (usually) tonic extension of the hindlimbs)	Pharmacodynamics of *theophylline*-induced seizures in rats [15] Chronic theophylline administration has no apparent effect on *theophylline* concentrations required to produce seizures in rats [92]^a^	Effect of infusion rate on *pentylenetetrazol* concentrations in serum, brain and cerebrospinal fluid of rats at onset of convulsions [34]

Most titles in this table are shortened titles. Full titles of the published papers include Kinetics of Drug Action in Disease States, followed by a number (I-XLV), and the short title represented in this table

^a^Titles are the full title of the published paper

### Enantiomers

As indicated above, another potentially confounding factor is enantio-selectivity, in particular if these drugs are administered as their racemic mixtures. In this respect it was appreciated that racemic mixtures are in principle mixtures of two drugs which differ in pharmacokinetics and pharmacodynamics (both qualitatively and quantitatively). Consequently the effect of a racemic drug depends on the interactions between the two enantiomers. To study such interactions properly, enantio-selective assays are needed, which allow measurement of the concentrations of each of the enantiomers separately [23]. This also applies when the effect of a single enantiomer is studied, as interconversion cannot a priori be excluded. To avoid complexities caused by enantio-selective disposition Levy and his associates were careful in the selection of the model drugs for their investigations. For example in the studies on the anesthetic effects, phenobarbital and heptabarbital were chosen as model drugs rather than the widely used thiopental [24].

### (Inter)active metabolites

There are numerous examples where active metabolites contribute to or are even entirely responsible for the effect following drug administration. With regard to the contribution of drug metabolites to the pharmacological effect, it was emphasized that metabolites can not only contribute to the effect through their intrinsic pharmacological activity, but that metabolites can also be inter-active (e.g. can compete with the parent drug for binding to target site while having no intrinsic activity). For this reason it is important that, when the pure metabolite is available, its effects are studied in combination with the parent drug at a relevant concentration range [11].

In the first paper we applied this concept to the possible interaction of the metabolite *p*-hydroxy-phenobarbital to the sedative effects of the parent drug phenobarbital [5]. In healthy animals no measurable concentrations of *p*-hydroxy-phenobarbital above the limit of quantification were observed at onset of LRR during a zero-order infusion of phenobarbital, nor at the offset of LRR following administration of an intravenous bolus dose of 140 mg/kg phenobarbital. Furthermore, upon direct administration of *p*-hydroxy-phenobarbital (0.0824 mg/min) in combination with its parent drug (0.824 mg/min), measurable concentrations in blood and CSF were detected, but there was no effect on the concentration of phenobarbital at onset of LRR. These results showed that there was no interaction between phenobarbital and its major metabolites [5]. Using a similar approach as for phenobarbital, Klockowski and Levy found that for diazepam the concentrations in CSF reflect the concentrations in the biophase [11]. The CSF concentrations could therefore be used to estimate the relative potency of the benzodiazepines and their respective metabolites, which was about 2:3:1:1 for diazepam and its metabolites desmethyldiazepam, temazepam and oxazepam. In addition, it was found that upon the administration of diazepam, the metabolite desmethyldiazepam contributes substantially to the hypnotic activity of the parent drug, despite its relatively minute concentration in serum [11].

A potential problem in studying the role of (inter)active metabolites is the fact the full metabolite profile of a given drug is rarely known. In this respect it is important that the possible role of (unknown) active metabolites may be explored indirectly by varying the rate and route of administration as has been elegantly demonstrated for heptabarbital [25]. Here oral administration is particularly informative in cases where there is a significant first-pass effect as this typically leads to large differences in the exposure to metabolites relative to the concentrations of the parent drug. Classical examples in which a difference in concentration–effect curves was observed after different routes of administration are quinidine [26], and verapamil [27, 28]. Meanwhile, changing the rate and route of administration and characterizing the pharmacodynamics upon the administration of different doses has become an accepted approach in the validation of PK–PD models as is illustrated for midazolam [29], alfentanil [30], and N^6^ cyclopentyl-adenosine [31].

### Acute tolerance as a potential complicating factor

Acute tolerance to drugs can develop very rapidly. Since it cannot be ruled out that the rate and/or extent of acute functional tolerance is not affected by disease conditions, it is necessary to minimize or avoid acute tolerance in the experimental setting [11, 32]. Here an important factor is that the drug *effect* rather than the drug concentrations may be the driver of pharmacodynamic tolerance development. For example, for barbiturates it was observed that the magnitude of acute tolerance was related to the maximum drug concentration at the site of action or the maximum intensity of the pharmacological effect, rather than the duration of exposure [33]. Therefore in Levy’s investigations, focus was placed on the drug concentration at the onset of effect rather than the offset, as in that situation the duration of the effect is equal to zero (see for example: [5, 11, 34]). In the first paper the development of acute tolerance development to phenobarbital under healthy conditions was studied by comparison of the concentrations at onset of LRR during a zero-order infusion relative to the concentrations at offset of LRR following administration of a bolus dose. These concentrations were equal, confirming that tolerance development had not occurred within the timeframe of the studies [5].

In summary, in the series of publications “Kinetics of Drug Action in Disease States” Levy and his associates have developed several concepts and approaches to separate pharmacokinetic from pharmacodynamic variability, which continue to be of value in developing pharmacokinetic-pharmacodynamic models.

## Exploring variability in pharmacodynamics

The concepts discussed above were applied to explore inter-individual variation in pharmacodynamics. The results of these studies are summarized in the Tables 2, 3, and 4. Levy started at a very early stage with exploring the effects of physiological variables on the pharmacodynamics. A summary of the various physiological factors and their effects on the pharmacodynamics of CNS active drugs that have been studied is presented in Table 2. The studies on the effects of disease on the pharmacodynamics focused on renal failure, liver failure, diabetes, hypertension (Table 3). As a rule, in these investigations the changes in pharmacodynamics were studied in two different models of a given disease, to exclude a possible artifact caused by the methods that had been used to introduce the disease, rather than the disease per se. Furthermore, extensive serum biochemistry was obtained to confirm the presence and the severity of the disease and to exclude to the best of our ability, co-morbidity. In these investigations, the most profound changes in brain sensitivity were observed in experimental renal failure [13, 16, 35–37].

**Table 2:** Influence of abnormal physiological conditions on the pharmacodynamics of CNS depressants and CNS stimulants

	CNS depressant drugs	CNS stimulating drugs
Pregnancy	Effect of pregnancy on *phenobarbital* concentrations at onset of loss of righting reflex in rats [93] Effect of pregnancy on *ethanol* concentrations at onset of loss of righting reflex in rats [10]^a^	Effect of pregnancy on the relationship between *phenytoin* concentration and antiseizure activity in rats [94]
Body temperature, fever	Effect of experimental fever on *phenobarbital* concentrations at onset of loss of righting reflex in rats [95]	Effect of fever on the pharmacodynamics of *theophylline*-induced seizures in rats [96] Effect of body temperature on the convulsant activity of *pentylenetetrazol* in rats [97]
Acute hypovolemia	Effect of acute hypovolemia on the pharmacodynamics of *phenobarbital* in rats [98] Effect of experimental hypovolemia on the pharmacodynamics and pharmacokinetics of *desmethyldiazepam* [99] Effect of hypovolemia on the pharmacodynamics of *zoxazolamine* in rats [100]	Effect of acute hypovolemia on *theophylline*-induced neurotoxicity in rats [101]
Food or fluid imbalances	Effects of acute starvation on the pharmacodynamics of *phenobarbital*, *ethanol* and pentylenetetrazol in rats and effects of refeeding and diet composition [102]^b^ Effects of acute fluid overload and water deprivation on the hypnotic activity of *phenobarbital* and the neurotoxicity of theophylline in rats [103]^b^	Effects of acute starvation on the pharmacodynamics of phenobarbital, ethanol and *pentylenetetrazol* in rats and effects of refeeding and diet composition [102]^b^ Effects of acute fluid overload and water deprivation on the hypnotic activity of phenobarbital and the neurotoxicity of *theophylline* in rats [103]^b^
Adrenalectomy, corticosterone treatment	Effect of adrenalectomy on the hypnotic activity of *phenobarbital*, the neurotoxicity of theophylline and pain sensitivity in rats [104]^b^	Effect of adrenalectomy on the hypnotic activity of phenobarbital, the neurotoxicity of *theophylline* and pain sensitivity in rats [104]^b^
Nicotine	Effect of nicotine on the pharmacodynamics and pharmacokinetics of *phenobarbital* and *ethanol* in rats [105]	
Drug-drug interactions	Effect of cyclosporine on the pharmacodynamics and pharmacokinetics of a barbiturate (*heptabarbital*) in rats [106] Potentiating effect of l-tryptophan on the hypnotic action of *phenobarbital* and *ethanol* in rats [107] Effects of contraceptive steroids on the pharmacodynamics of *ethanol* in rats [108]	

Most titles in this table are shortened titles. Full titles of the published papers include Kinetics of Drug Action in Disease States, followed by a number (I-XLV), and the short title represented in this table

^a^Titles are the full title of the published paper

^b^If both CNS depressants and CNS stimulants are reported in one published paper, the paper is mentioned twice in the table

**Table 3:** Influence of disease conditions on the pharmacodynamics of CNS depressants and CNS stimulants

	CNS depressants	CNS stimulants
Renal dysfunction	Effect of experimental renal dysfunction on *phenobarbital* concentrations in rats at onset of loss of righting reflex [35] Effect of experimental renal dysfunction on the pharmacodynamics of *ethanol* in rats [36] Effect of experimental renal failure on the pharmacodynamics of *zoxazolamine* and *chlorzoxazone* [13] Effect of orally administered activated charcoal on the hypnotic activity of *phenobarbital* and the neurotoxicity of theophylline administered intravenously to rats with renal failure [109]^b^	Effect of experimental renal failure on the pharmacodynamics of *theophylline*-induced seizures in rats [37] Disparate effects of *pentylenetetrazol* in rats as a function of renal disease model and pharmacologic endpoint [16] Effect of orally administered activated charcoal on the hypnotic activity of phenobarbital and the neurotoxicity of *theophylline* administered intravenously to rats with renal failure [109]^b^
Liver disease	Effect of experimental liver diseases on the pharmacodynamics of *phenobarbital* and *ethanol* in rats [110] Effect of hepatic cirrhosis on the pharmacodynamics and pharmacokinetics of *mivacurium* in humans [111]^a^	Effect of experimental liver disease on the neurotoxicity of *theophylline* in rats [112]
Diabetes	Effect of experimental diabetes on *phenobarbital* concentrations in rats at onset of loss of righting reflex [113]	
Hypertension	Effect of experimental hypertension on the pharmacodynamics of *phenobarbital* in rats [114]	
Hyperthyroidism	Effect of experimental hyperthyroidism on the hypnotic activity of a benzodiazepine (*oxazepam*) in rats [12] Effect of experimental thyroid disorders on the pharmacodynamics of *phenobarbital*, *ethanol* and pentylenetetrazol [115]^b^	Effect of experimental thyroid disorders on the pharmacodynamics of phenobarbital, ethanol and *pentylenetetrazol* [115]^b^

Most titles in this table are shortened titles. Full titles of the published papers include Kinetics of Drug Action in Disease States, followed by a number (I-XLV), and the short title represented in this table

^a^Titles are the full title of the published paper

^b^If both CNS depressants and CNS stimulants are reported in one published paper, the paper is mentioned twice in the table

**Table 4:** Influence of systemic components of renal dysfunction on the pharmacodynamics of CNS depressants and CNS stimulants

CNS depressants	Effect of dialyzable component(s) of uremic blood on *phenobarbital* concentrations in rat at onset of loss of righting reflex [42] Acute effect of urea infusion on *phenobarbital* concentrations in rats at onset of loss of righting reflex [24] Effect of elevated plasma creatinine concentrations on the hypnotic action of *phenobarbital* in normal rats [116] Effect of experimental nephrotic syndrome on the pharmacodynamics of *heptabarbital*: Implications of severe hypoalbuminemia [43]
CNS stimulants	Effect of the dialyzable component(s) of uremic blood on *theophylline* neurotoxicity in rats [117]

Titles in this table are shortened titles. Full titles of the published papers include Kinetics of Drug Action in Disease States, followed by a number (I-XLV), and the short title represented in this table

The effect of renal dysfunction was studied by using two distinctly different animal models of renal failure; uranyl nitrate was used to chemically induce renal dysfunction and bilateral ureteral ligation was used to mechanically induce renal dysfunction [13, 16, 35–37]. In rats with experimental renal failure, significantly increased brain sensitivity for the sedative effect of phenobarbital was found, as reflected by a lower threshold dose for induction of LRR and lower phenobarbital concentrations in plasma, brain tissue and CSF at onset of LRR [35].

Given the importance of this observation, the mechanism(s) of this change were explored in a number of subsequent studies (Table 4). Since it could not be excluded a priori that (severe) renal failure is associated with a change in target site distribution, resulting from changes in the permeability of the blood–brain barrier, and/or the expression and function of transporters at the blood–brain barrier, the target site distribution kinetics were revisited. To this end the influence of the infusion rate on the phenobarbital plasma, tissue and CSF concentrations at onset of LRR was determined in rats with experimental renal failure. These studies showed that the kinetics of biophase equilibration was indeed altered in renal failure, since, unlike the situation in normal rats, the CSF concentration at onset of LRR changes with the infusion rate [35]. However, at each infusion rate the concentration at LRR was lower in renal failure rats, compared to their healthy controls. On the basis of extrapolation to a hypothetical infusion rate of zero, the change in brain sensitivity was confirmed and the true magnitude of this change could be quantified [35].

Following a “systems approach” the next step was to determine whether changes in brain sensitivity in experimental renal failure are also observed for drugs with a similar, a related or a different mechanism of action. For example, it is well established that the actions of barbiturates originate at least in part from an interaction with GABA receptor complex [38, 39]. For ethanol on the other hand, the sedative effect is at best only in part related to the GABA receptor [36, 40]. Interestingly, for the barbiturate heptabarbital a similar change in brain sensitivity was observed compared to phenobarbital [41]. For ethanol also increased brain sensitivity was observed, albeit that the magnitude of this change was smaller than for barbiturates [36]. For the muscle relaxant zoxazolamine, with a distinctly different mechanism of action, an increased brain sensitivity was also found in rats with renal failure [13]. These findings indicate that in general renal failure is associated with an increased sensitivity to the actions of sedative and anesthetic drugs, which is not related to a specific mechanism of action.

To mechanistically understand the observed changes in pharmacodynamics, Levy was particularly keen on studying the effects of endogenous compounds which accumulate in the body in renal failure and which alter the pharmacodynamics causing the increased brain sensitivity (Table 4). In the search for the identification of such mediators, endogenous components from uremic plasma were isolated by dialysis. Next the effect of dialyzable components from the blood of uremic rats was studied by administering the dialysate to normal rats and then to study the pharmacodynamics of phenobarbital by determining the CSF concentration at onset of LRR [42]. This study showed a change in brain sensitivity very similar to the change observed in rats with experimental renal failure [42]. Next, a single component from the dialysate on the pharmacodynamics of barbiturates was studied, i.e. urea. Urea infusion resulted in experimental azotemia (i.e. blood urea nitrogen concentrations in the same range as in rats with experimental renal failure), and was found to affect the distribution kinetics of phenobarbital. However, the brain sensitivity was not changed as the CSF concentrations of phenobarbital at LRR were unaffected [24]. Finally it was demonstrated that nephrotic syndrome caused hypo-proteinemia and an associated decrease in plasma protein binding of heptabarbital (i.e. a change in pharmacokinetics) [43]. But again the drug concentration in CSF at the pharmacological endpoint was not changed, indicating that nephrotic syndrome has no effect on the pharmacodynamics.

The influence of renal failure was also studied for another pharmacodynamic endpoint: seizure activity as a measure of neurotoxicity. For the convulsant effect of PTZ, disparate effects of renal failure were observed, both with respect to the pharmacodynamic endpoint and the method that was used for induction of renal failure [34]. No difference in the concentration of PTZ required for induction of minimal seizures was observed between normal rats and rats with chemically or surgically induced renal failure. In contrast, rats with chemically induced renal dysfunction, required higher PTZ concentrations for the induction of maximum seizures, whereas the ureter-ligated rats convulsed at lower concentrations of PTZ than did the corresponding control animals [16]. It was suggested that renal failure may have differential effects on the threshold for induction of seizures and the spreading of the seizure activity.

The studies on the convulsant effects were extended to the (at that time) still widely used brochodilator theophylline. This drug can cause serious side effects, including life-threatening generalized seizures [44, 45]. Levy and his associates investigated the wide variability in theophylline plasma concentrations associated with seizures. To this end the theophylline concentration in the CSF at the onset of seizures, which was found to be independent of the rate of infusion, was used as pharmacodynamic endpoint [15]. Using different models to induce renal dysfunction, it was found that the neurotoxicity of theophylline was not changed after chemical induction of renal failure (uranyl nitrate), while a higher incidence of neurotoxicity was found after mechanical induction (ureter ligation) of renal dysfunction [37].

## The impact on pharmacodynamics research: towards physiology based pharmacodynamic models

In the previous paragraphs an account is presented of Levy’s research on variation in pharmacodynamics as described in the publications in the series “Kinetics of Drug Action in Disease States”. By applying experimental approaches that enable a strict separation between pharmacokinetics and pharmacodynamics it was shown unequivocally that besides variation in pharmacokinetics, variation in pharmacodynamics is a significant determinant of variation in drug response. Moreover, evidence of a wide disparity in the effects of disease on the pharmacodynamics of CNS active drugs was observed. However, the evidence that had been generated was still largely observational. Little was known on the underlying mechanisms of the observed changes. Moreover, it was still largely unknown how the observed variation in experimental models of disease, would translate into inter-individual variation in humans. The next challenge was therefore to develop the mechanism-based understanding of this variation in pharmacodynamics. Analogous to the development of the theory and application of “physiology-based pharmacokinetics” (PBPK) to explain and predict variation in drug *concentrations* [46, 47], it was deemed necessary to develop “physiology-based pharmacodynamics”(PBPD) as a novel scientific basis for the understanding and prediction of variation in drug *effects*.

**Fig. 2:**
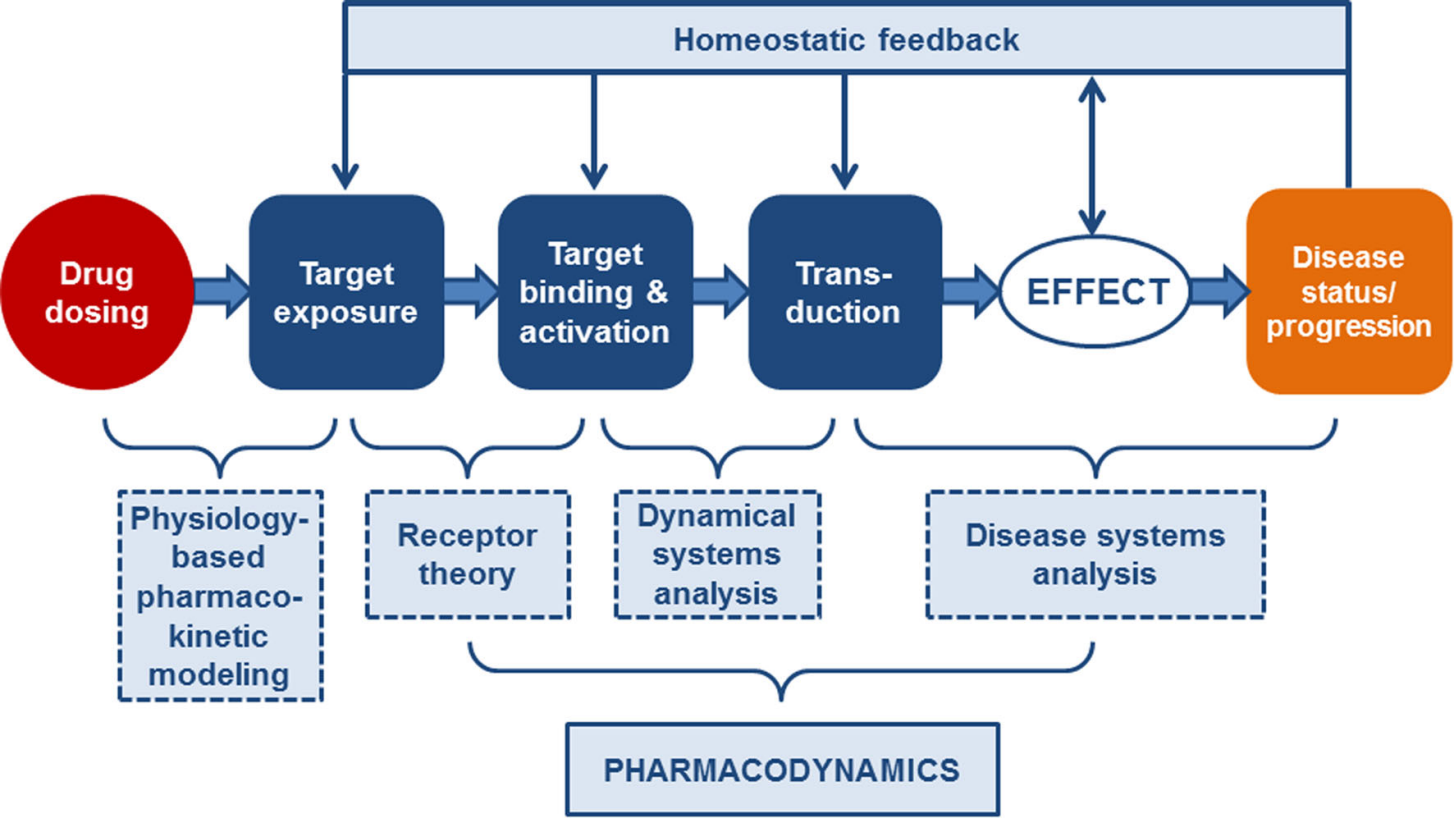
Schematic representation of physiology-based pharmacodynamic (PBPD) modeling. PBPD models connect pharmacokinetics to the drug effects on disease progression, and contain expressions to describe the processes on the causal path between drug administration and effect (target site distribution, target binding and activation, and transduction and homeostatic feedback)

PBPD models contain specific expressions to characterize in a strictly quantitative manner processes on the causal path between exposure (in terms of concentration at the target site) and the drug effect (in terms of the change in biological function). Pertinent processes on the causal path are: (1) target site distribution, (2) target binding and activation and (3) transduction and homeostatic feedback. PBPD models connect pharmacokinetics to ultimately the drug effects on disease progression (Fig. 2). As is the case for PBPK models, an important feature of PBPD models is the strict separation between drug specific properties (in terms of the binding and activation of the target) and system-specific properties (in terms of transduction processes).

To develop meaningful PBPD models it was necessary to overcome a number of technical challenges. Levy’s research was based on a single observation of the drug concentration and the effect intensity. However PBPK and PBPD focus on the modeling of the time course of drug concentrations and effect, respectively. Hence experimental methods needed to be developed that enabled multiple observations per individual subject. Second, continuous and meaningful measures of the pharmacological effect are needed to explore the full relation between drug concentration and effect intensity. Finally, computational techniques needed to be developed to identify complex, often non-linear, PBPD models.

### Target site distribution

In the original studies CSF samples were collected by puncture of the cisterna magna, limiting the number of samples per individual animal to one. To enable time course studies, the experimental technique of implantation of a permanent cannula in the cisterna magna was developed. The first study showing the feasibility of time course studies in CSF focused on heptabarbital [48]. Next the technique was applied to study the CNS distribution of desglycinamide-arginine vaspressin (DGAVP) as prototype peptide [49]. By applying the “unit impulse response” technology, analyzing the concentration profiles following intravenous and intracerebral administration, the input profile in the CNS could be determined [50]. Using this technology, the brain distribution kinetics of the enantiomers of baclofen was determined, showing remarkable differences in the brain distribution kinetics between the enantiomers [51]. Comparison of the input profile obtained on the basis of CSF concentrations with the ones obtained on the basis of EEG effect parameters as a pharmacodynamic endpoint showed markedly different input profiles indicating that CSF concentrations of baclofen are not pharmacokinetically identical to the target site concentrations [52]. A systematic review of this topic indeed showed that the use of CSF concentrations is limited in predicting the effect of a centrally acting drug, presumably as a result of compartmentalization within the brain [53]. Recent studies, using microdialysis to determine drug concentration profiles, have shown that brain distribution kinetics are complex and often non-linear due to regional differences blood–brain barrier permeability as well as regional differences in the expression and function of transporters at the blood–brain barrier [54–56]. Meanwhile, the first PBPK models for characterization drug distribution in the central nervous systems have been proposed, for model drugs paracetamol, quinidine, and methotrexate [55, 57, 58]. It is anticipated that PBPK concepts will increasingly be applied to characterize target distribution kinetics [57].

### Target binding and activation

Fundamental research on the mechanisms of variation in pharmacodynamics is based on the analysis of full in vivo concentration–effect relationships. This requires the availability of continuous measures of the pharmacological effect, which can be obtained continuously/repeatedly within individual subjects and which are meaningful with regard to the therapeutic effects and/or the safety of the drug under investigation [59]. A major development in the research on pharmacodynamics was the creation of chronically instrumented animal models in which, for a variety of endpoints, the time course of the drug effect could be determined in conjunction with the time course of the drug concentration in blood plasma. This enabled the derivation of concentration–effect relations in individual animals for a variety of drugs (Table 5). Following Levy’s approach, for prototype compounds from each of these classes (i.e. midazolam, alphaxalone, N^6^-cyclopentyl adenosine, alfentanil, remoxipride) it was demonstrated that unique concentration–effect relations had been obtained that were independent of the rate and route of administrations [60] [29–31] [61]. In contrast for the 5-HT_1A_ receptor agonists buspirone, it was demonstrated that the active metabolite 1-(2-pyrimidinyl)-piperazine contributes significantly to the effect following administration of the parent drug [62]. Furthermore, for benzodiazepines and 5 HT_1A_ receptor agonists the role of interactive metabolites was studied by direct administration [63] [64], while for synthetic opioids and dopamine D_2_ receptor antagonists, the analysis of the effect upon repeated administration enabled the characterization of acute functional tolerance development [30, 61].

**Table 5:** Overview of studies in which a continuous measurement of the pharmacological effect was used together repeated measurement of pharmacokinetics

Drug	Endpoint
Benzodiazepines + related GABA receptor agonists	EEG parameters [29, 60, 66]
Cyclopentyl-adenosine A_1_ receptor agonists	Hemodynamic parameters [31] Biochemical parameters: lipolysis [118]
Opioids	EEG effect parameters [119, 120] Anti-nociceptive effect; Respiratory depression [71, 121]
5-HT_1A_ receptor agonists	Body temperature [64, 83]
Dopamine D_2_ receptor agonists	Receptor occupancy [122, 123] Prolactin responses [61]

**Fig. 3:**
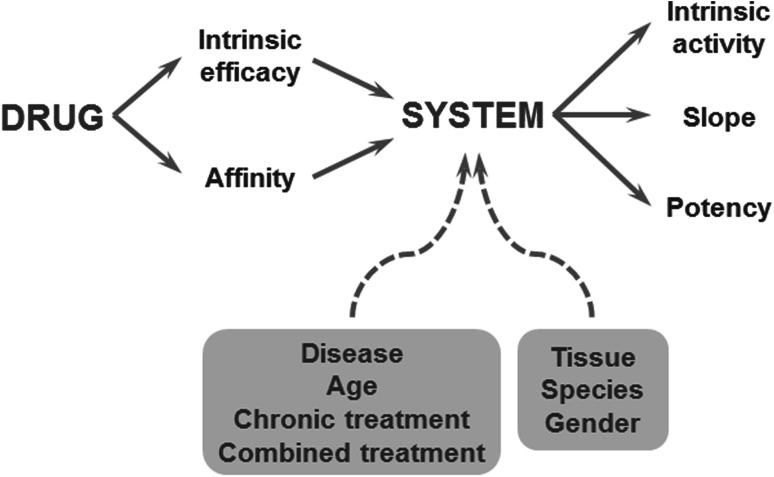
The relationship between drug concentration and the intensity of the biological response depends on drug- and biological system specific factors. Drug specific properties are the target binding affinity and the intrinsic efficacy, which govern the target activation. A biological system-specific transducer function describes the relation between the target activation and the effect. Reproduced from: van der Graaf and Danhof [67]

**Fig. 4:**
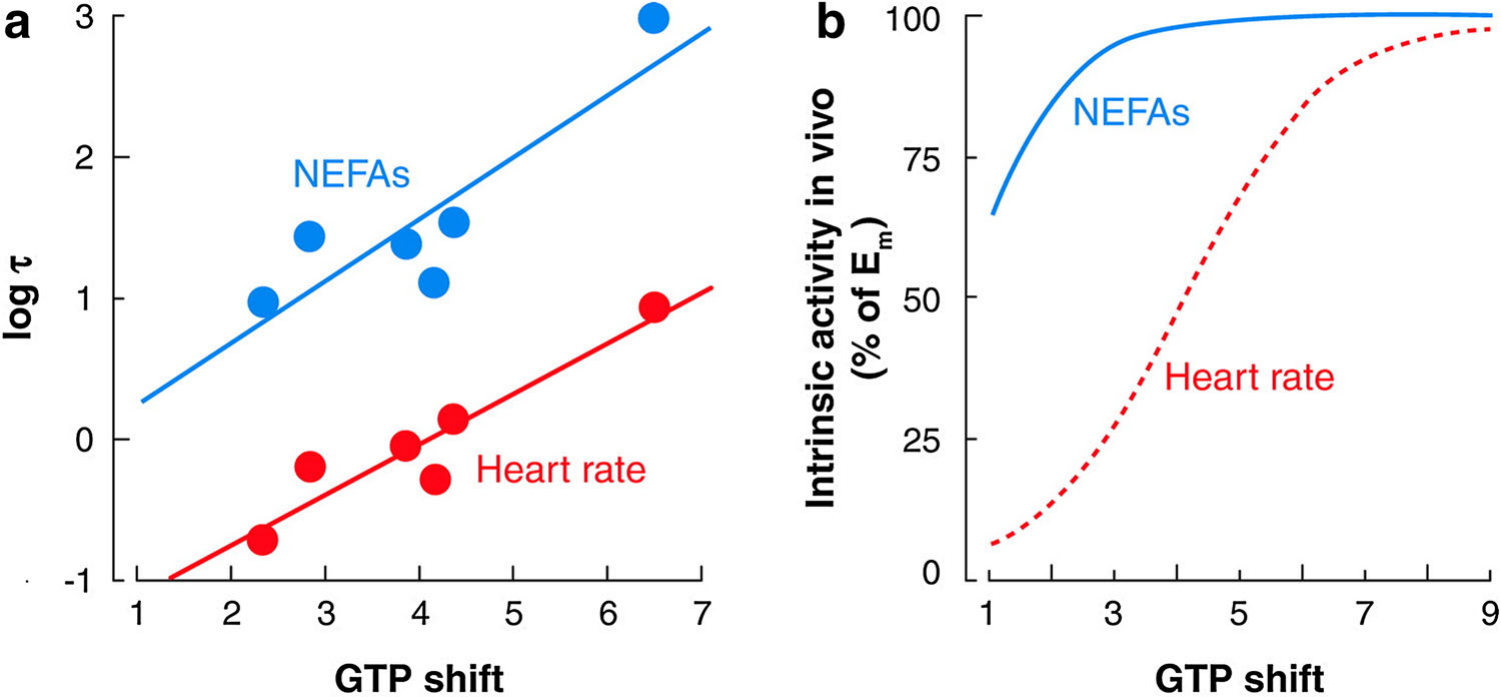
PK–PD modeling of anti-lipolytic effects of Adenosine A1 receptor agonists in rats: prediction of tissue-dependent efficacy in vivo. **a** Relationship between intrinsic efficacy in an in vitro (GTP-shift) and in vivo (log *τ)* bioassay for the effect of a series of A1 receptor agonists on heart rate and lipolysis (as measured by nonesterified fatty acids, NEFAs), respectively. The difference in the intercept for the two effects is explained by the difference in receptor density between adipose tissue and cardiac tissue. **b** Relationship between intrinsic efficacy in an in vitro bioassay (GTP shift) and in vivo intrinsic activity (*α*) for the effects on heart rate and lipolysis, respectively. The *graphs* show that partial agonists with GTP shift values between 1 and 5 display the highest selectivity of action for the effect lipolysis versus heart rate. Reproduced from van der Graaf et al. [70]

**Fig. 5:**
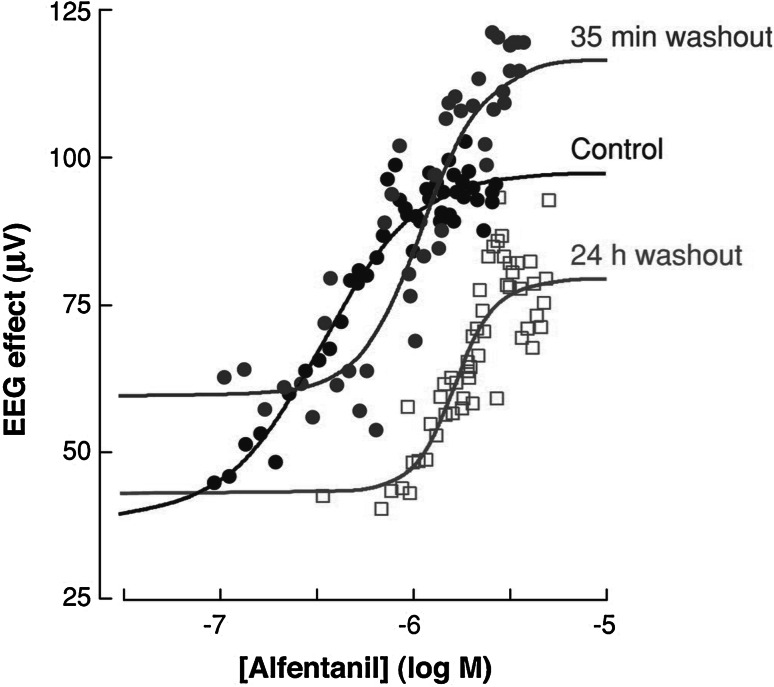
PK–PD analysis of the EEG effect of alfentanil in rats following in vivo μ-opioid receptor (MOP) knockdown with β-flunaltrexamine. Pretreatment with β-flunaltrexamine resulted in an approximately 60 % reduction of functional MOP receptors at 35 min and at 24 h post administration. A parallel shift in the concentration–effect relationship without a major change in maximum effect was observed. This reduction in functional receptors is consistent with the observation that the MOP receptor functions with a high receptor reserve. Reproduced from Garrido et al. [74]

A milestone in the research on pharmacodynamics was the incorporation of concepts from receptor theory for the prediction of variation in concentration–effect relations [65]. In theory, the relationship between the drug concentration and the intensity of the biological response depends on drug- and biological system specific factors (Fig. 3). This explains why for a given drug, the concentration–effect relationship can differ between tissues, between species and, within a single species, also between individuals. Classical receptor theory combines two independent parts to describe drug action: an agonist-dependent part and a system dependent part and therefore constitute a unique scientific basis for the prediction of variation in in vivo concentration–effect relationships Briefly, the agonist dependent part describes the target activation, usually on the basis of a hyperbolic function. The target activation depends on the intrinsic efficacy of the drug under investigation and the receptor density. Next the system dependent part describes the translation of the target activation into the response on the basis of a system-specific transducer function. This transducer function can take any shape (i.e. linear, hyperbolic) [65]. In a number of investigations it was shown that for a given target, receptor models can be identified by simultaneously analyzing concentration–effect relations of a training set of ligands with different binding affinity and intrinsic efficacy, yielding estimates of the in vivo binding affinity and intrinsic efficacy of each of the drugs in the training set, as well as the shape and location of the system specific non-linear transducer function. For GABA_A_-receptor agonists, adenosine A_1_ receptor agonists, (semi-) synthetic opioids and 5-HT_1A_ receptor agonists highly significant correlations were observed between the affinity and intrinsic efficacy estimates in vivo and corresponding estimates in in vitro bioassays confirming the validity of the approach [64, 66–68]. Thereby it was demonstrated that for individual compounds, in vivo drug concentration–effect relationships can be predicted on the basis of information from these in vitro assays, provided that the effects of potentially confounding pharmacokinetic factors are either excluded or accounted for, as was demonstrated for the 5-HT_1A_ receptor agonist flesinoxan, which has a much lower in vivo potency than expected on the basis of its receptor affinity, due to active efflux mechanisms at the blood–brain barrier [69]. Successful applications of receptor theory include prediction of the selectivity of action of N^6^ cyclopentyladenosine analogues (inhibition of lipolysis *versus* bradycardia [70]; Fig. 4), prediction of the selectivity of action of semi-synthetic opioids (anti-nociception versus respiratory depression [71]), prediction of concentration–effect relations of (semi-) synthetic opioids in man [72, 73], and the prediction of variation in concentration–effect relations of alfentanil (Fig. 5) [74].

### Transduction and homeostatic feedback

In PK-PD modeling the concept of ‘transduction’ refers to the processes that govern the transduction of target activation into the response in vivo. Modeling of transduction can be complex because it is typically highly non-linear. Also, complex homeostatic feedback mechanisms and/or compensatory pathways might attenuate or alter the response of the in vivo concentration–effect relationship [75–77]. When transduction in vivo is slow (i.e. operating at rate constants in the order of minutes to hours, or even days) transduction will also determine the time course of drug effect. Moreover, in this situation homeostatic feedback mechanisms might cause complex patterns of the pharmacodynamics, such as fluctuating (oscillating) pharmacological effect *versus* time profiles, dependency of drug effects on the rate of administration, tolerance development upon chronic treatment and/or rebound effects upon cessation of chronic treatment. This underscores the importance of the modeling of time-dependent transduction mechanisms. To account for time-dependent transduction in PK-PD modeling, a variety of models have been proposed that are all based on concept of the indirect pharmacological response model that was proposed by Levy in 1969 to account for the observed delay in the anticoagulant response of warfarin [7]. This concept has been formalized in a large series of publications by Jusko et al. [78–81]. Turnover models can also be linked in the sense that the output of one model serves as input for another. Using these cascading turnover models, intermediary processes between the drug–target interaction and the ultimate biological response can be described; this approach has been applied to modeling the effects of corticosteroids on the activity of the enzyme tyrosine amino transferase (TAT) [82]. In addition, the effect versus time profiles of the hypothermic response can also be described with a complex turnover model [64]. Together with allometric scaling the hypothermic response of two selective 5-HT_1A_ receptor agonists, buspiron and ipsapirone, could be predicted in humans [83]. This study showed that the importance of scaling factors in the interspecies extrapolation of drug effects [84].

### Disease systems analysis

Levy’s research focused on the effects of disease on drug action (i.e. on disease as a factor causing inter-individual variation in drug response). However, drug treatment may also have an effect on the disease. In many instances drug effects are symptomatic, but drugs may also modify disease process and thereby modify disease progression. This is particularly important for chronic progressive diseases. Traditionally, descriptive models have been used to characterize drug effects on disease progression [85, 86]. Recently the concept of disease systems analysis has been introduced, which aims to describe disease progression in a more mechanistic manner. Disease systems analysis is based on the “turnover model” concept to characterize variation in the rate of disease progression. A pertinent feature of disease systems models is the strict separation between drug effects on the disease status (i.e. symptomatic effects) *versus* drug effects on the disease process (disease modifying effects) [84, 87]. In a series of simulations it has been demonstrated that depending on the target and the mechanism of action, these disease progression models have distinctly different signature profiles, enabling the distinction between symptomatic and disease modifying effects [88]. Meanwhile cascading turnover models (i.e. models in which the output from one turnover model serves as input for a second turnover model) have been proposed to cope with different time scales in disease progression analysis [89]. This approach has been successfully applied to the characterization of disease progression in type-2 diabetes mellitus and osteoporosis [88, 90].

## Conclusion

In this paper an overview has been given of Gerhard Levy’s contributions to research on the interrelationships between pharmacokinetics and pharmacodynamics in the 50 years following the publication of his seminal paper on the kinetics of pharmacologic effects in 1966 [6]. It is shown how the introduction of the theoretical concepts on the “kinetics of pharmacological effects”, the introduction of the “turnover model”, and ultimately the experimental research on “the kinetics of drug action in disease”, has yielded the scientific basis for the development of “Physiology-Based PharmacoDynamic” (PBPD) modelling as novel scientific discipline, which in concert with “Physiology-Based PharmacoKinetic” (PBPK) modelling constitutes the theoretical basis for “Physiology-Based Pharmacokinetic & Pharmacodynamic (PBPKPD) modeling a “systems approach” to the prediction of drug effects. PBPKPD modelling connects observed drug concentrations in plasma to the effects on disease progression. This will enable the unravelling the complex interrelationships between drug action and disease progression.
